# Intergenerational Financial Support and Mental Health: A Comparison of Welfare Policy Contexts in Europe

**DOI:** 10.1093/geroni/igaf122.1316

**Published:** 2025-12-31

**Authors:** Kent Jason Cheng, Ariel Azar, Morten Wahrendorf

**Affiliations:** University of Massachusetts Boston, Boston, Massachusetts, United States; Purdue University, West Lafayette, Indiana, United States; Heinrich-Heine-University Düsseldorf, Düsseldorf, Nordrhein-Westfalen, Germany

## Abstract

As Europe faces a rapidly aging population, understanding the interplay between family and welfare state support becomes critical for addressing mental health concerns among older adults. This study examines the association between financial transfers from descendants and depressive symptoms, exploring variations across European welfare state contexts with differing levels of pension generosity and gender differences in the associations explored. Using data from 20 European countries included in the Survey of Health, Ageing, and Retirement in Europe (SHARE), waves 3–9, we examined predictors of depressive symptoms measured by the EURO-D scale. Key predictors included receiving financial transfers of EUR 250 or more from children or grandchildren, the net replacement rate of pensions, and the interaction between these variables. Receiving financial support from children or grandchildren is associated with higher depressive symptoms, signalling that the transfer often arises due to the older adult’s need. Gender differences were notable: men experienced a greater increase in depressive symptoms when receiving transfers, likely due to their stronger ties to labour force participation and the impact of pension adequacy. Robust welfare states amplified the association between receiving transfers and depressive symptoms, highlighting a complex dynamic between state support and family assistance. This may reflect situations where dual sources of support indicate severe underlying needs. These findings align with contingency exchange theories and crowding-in, emphasizing that family and the welfare state often work in tandem rather than cancelling each other out to address significant hardships faced by the most vulnerable segments of the population.

